# Paramedic-delivered teleconsultations: a grounded theory study

**DOI:** 10.1007/s43678-021-00224-6

**Published:** 2021-12-07

**Authors:** Richard Armour, Jennie Helmer, John Tallon

**Affiliations:** 1 British Columbia Emergency Health Services, 2955 Virtual Way, Vancouver, BC V5M 4X6 Canada; 2 School of Health and Related Research, University of Sheffield, Sheffield, UK; 3 Paramedic Academy, Justice Institute of British Columbia, New Westminster, BC Canada; 4 School of Population and Public Health, University of British Columbia, Vancouver, BC Canada; 5 Department of Emergency Medicine, University of British Columbia, Vancouver, BC Canada

**Keywords:** Paramedic, Medical support, Medical control, Peer support, Clinical advice, Prehospital

## Abstract

**Objective:**

Progression in Anglo-American models of out-of-hospital care has resulted in the development of alternative roles for paramedics, including advanced paramedics providing teleconsultations to frontline paramedics. Traditionally provided by physicians, little is known about how paramedics perceive peer-to-peer teleconsultations. This research aimed to explore paramedic perceptions of paramedic-delivered teleconsultations.

**Methods:**

This investigation employed a constructivist grounded theory methodology. Six focus groups were conducted with purposive and theoretical sampling and data analyzed using open coding and continual comparative analysis.

**Results:**

33 paramedics from across British Columbia, Canada, participated in the focus groups. Seven key themes emerged during the focus groups; the perceived roles and status of paramedic specialists and physicians in healthcare, the influence of relationships and culture on clinical consultations, practicalities of out-of-hospital care and the importance of lived experience, provision of appropriate clinical advice, professional trust and respect, mentorship in out-of-hospital care and clinical governance and education requirements. This led to the development of the grounded theory *paramedics increasing ownership of their profession*.

**Conclusion:**

Paramedics reported a number of areas in which paramedic-delivered teleconsultations provided benefits not seen with traditional physician-delivered teleconsultation model. Emergency health systems delivering an Anglo-American model of care should consider the possible benefits of paramedic-delivered teleconsultations.

**Supplementary Information:**

The online version contains supplementary material available at 10.1007/s43678-021-00224-6.

## Résumé

**Objectif:**

La progression des modèles anglo-américains de soins extrahospitaliers a donné lieu à l’élaboration d’autres rôles pour les ambulanciers paramédicaux, y compris des ambulanciers paramédicaux avancés qui offrent des services de téléconsultation aux ambulanciers paramédicaux de première ligne. Traditionnellement fournis par les médecins, on sait peu de choses sur la façon dont les ambulanciers paramédicaux perçoivent les téléconsultations entre pairs. Cette recherche visait à explorer les perceptions des paramédicaux sur les téléconsultations effectuées par les paramédicaux.

**Méthodes:**

Cette enquête a utilisé une méthodologie constructiviste de théorie ancrée. Six groupes de discussion ont été menés avec un échantillonnage raisonné et théorique et les données ont été analysées en utilisant un codage ouvert et une analyse comparative continue

**Résultats:**

33 ambulanciers paramédicaux de toute la Colombie-Britannique, Canada, ont participé aux groupes de discussion. Sept thèmes clés ont émergé au cours des groupes de discussion : la perception des rôles et du statut des spécialistes paramédicaux et des médecins dans les soins de santé, l'influence des relations et de la culture sur les consultations cliniques, les aspects pratiques des soins extrahospitaliers et l'importance de l'expérience vécue, la fourniture de conseils cliniques appropriés, la confiance et le respect professionnels, le mentorat dans les soins extrahospitaliers et les exigences en matière de gouvernance clinique et de formation. Cela a conduit au développement de la théorie ancrée des ambulanciers paramédicaux augmentant l'appropriation de leur profession.

**Conclusion:**

Les paramédicaux ont signalé un certain nombre de domaines dans lesquels les téléconsultations paramédicales offraient des avantages qui n'avaient pas été constatés avec le modèle traditionnel de téléconsultation par les médecins. Les systèmes de santé d'urgence appliquant un modèle de soins anglo-américain devraient tenir compte des avantages possibles des téléconsultations dispensées par des ambulanciers paramédicaux.

**Clinician’s capsule**

***What is known about the topic?***

Paramedic services utilize teleconsultations to provide advice to paramedics, historically provided by physicians, some are now provided by advanced paramedics.

***What did this study ask?***

This research aimed to explore the experience of paramedics receiving teleconsultations from advanced paramedics.

***What did this study find?***

Paramedics described a number of areas in which paramedic-delivered teleconsultations provided benefits that physician-delivered teleconsultations did not.

***Why does this study matter to clinicians?***

Paramedic-delivered teleconsultations remain in their infancy but appear to possibly provide benefits to paramedics and patients.

## Background

The out-of-hospital setting remains fraught with ambiguity, lacking diagnostic testing and imaging required to make definitive diagnoses. Compounding this is the isolated nature of paramedic work, with limited access to specialist consultation or support in times of indecision or crisis. To mitigate this risk many paramedic services provide access to emergency teleconsultation services [1–4]. This support has historically been provided by physicians and includes advanced clinical advice, transport decision-making and authorization for resuscitation discontinuation [1–4]. However, with the professionalization and self-regulation of paramedics and increasingly common undergraduate and postgraduate education for paramedics [5–7], paramedic services have recognized the role paramedics may play in delivering teleconsultations and developed such services [8–10].

British Columbia Emergency Health Service (BCEHS) is the largest paramedic service in Canada, covering 944,000km^2^ and responding to over 700,000 9-1-1 calls each year [11]. BCEHS provides an Anglo-American model of out-of-hospital care, utilizing paramedics in the delivery of clinical care [12]. The majority of care is delivered by emergency medical responders and primary care paramedics, supported by advanced care paramedics and critical care paramedics in higher acuity and more complex cases [12].

In 2016, BCEHS developed the CliniCall service in response to swelling service demand and increasingly complex clinical presentations driven by the opioid epidemic [13]. Based on national and international paramedic-delivered teleconsultation services [8–10], CliniCall provides 24/7 access to paramedic specialists with substantive clinical experience and backgrounds in higher education and advanced clinical practice functioning under the organizational model of physician oversight. Paramedic specialists are either advanced care paramedics or critical care paramedics and provide ad-hoc clinical advice and support, transport decision making guidance and authorization for the implementation of some complex clinical practice guidelines which require consultation prior to implementation [14].

Despite the increasingly widespread uptake of paramedic-delivered teleconsultations, little research examines the experience of paramedics receiving teleconsultations from peers. This research aimed to explore the experience of paramedics receiving peer-to-peer teleconsultations, the factors which may lead to a positive or negative experience and understand how this experience compares with traditional physician-based teleconsultations.

## Methods

### Research design

Grounded theory was employed in this project as given the paucity of available literature it is hoped moving beyond a superficial analysis of responses will assist in guiding future research across various settings [15, 16]. Constructivist grounded theory, based on interpretivist, subjectivist assumptions in which reality is constructed rather than discovered [17, 18] was chosen as it was anticipated the variety of backgrounds, education and practice environments across BC were unlikely to produce a singular, discoverable reality. This research was conducted and reported in accordance with COREQ guidelines.

### Sample

Emergency medical responder, primary care paramedic and advanced care paramedic staff within BCEHS most frequently engage with CliniCall and so members of staff holding these licenses with previous experience of both paramedic- and physician-delivered teleconsultations were purposively invited to participate. CliniCall staff were not invited to participate. Advertisements were placed in the weekly BCEHS bulletin, monthly clinical update and operational Facebook page along with the participant information sheet for recruitment. Sample size determination in qualitative research is less linear than in quantitative research [19], as it is driven by data saturation rather than data volume [19, 20]. A planned minimum sample of 30 participants was sought in accordance with recommendations for grounded theory [21].

### Data collection and storage

Focus groups were utilized in this research to provide a framework for discussion whilst allowing interpersonal engagement between respondents [22]. These focus groups were conducted and recorded within Zoom (Version 5.0.3) by RA using a pre-determined guide (Supplemental Appendix 1). The focus groups were transcribed verbatim and transferred to NVivo for analysis. Data was managed in accordance with the Canadian Institute of Health Research (CIHR) standards [23].

### Data analysis

Data analysis was conducted in NVivo by RA using an initial phase of open coding after each focus group followed by focussed coding. Constant comparative analysis was used to identify emerging categories and themes and to assist with theoretical sampling of participants during later groups.

### Academic rigor

Guba & Lincoln’s criteria of credibility, transferability, dependability and confirmability was used [24]. Credibility was addressed by analyst triangulation, with author JH confirming the coding was grounded in the obtained data. Transferability has been enhanced by providing descriptions of the practice environment the research was conducted in. Dependability was improved using a well-recognized method of qualitative research and maintaining an audit trail. Confirmability and reflexibility was crucial in this research given the primary author’s previous experience as an advanced care paramedic and colleague, and so the research proposal and interview guide were reviewed by an independent committee of paramedics and physicians for review and comments, whilst active reflection during coding was performed.

### Ethics

Ethics approval was granted by the University of Sheffield, BCEHS research and evaluation subcommittee and the University of British Columbia research ethics board.

## Results

### Participant recruitment and preliminary results

**Fig. 1:**
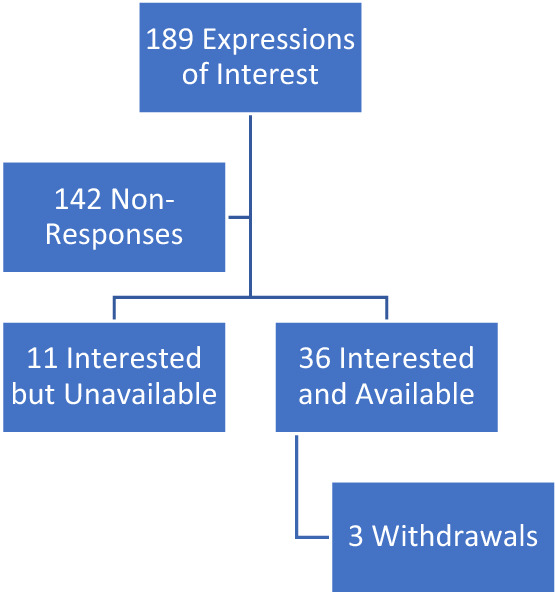
Participant recruitment

Participant recruitment and characteristics are summarized in Fig. 1 and Table 1. One pilot group was conducted to evaluate the interview guide, with two subsequent purposively sampled groups and three theoretically sampled groups before achieving theoretical saturation. During preliminary open coding 232 unique codes were identified which were distilled into 73 unique focussed codes (Supplemental Appendix 2). Seven key themes ultimately emerged from the data.

**Table 1:** Participant characteristics

Sex	Male: 18/33 (55%)
	Female: 15/33 (45%)
License level	Emergency medical responder: 2/33 (6%)
	Primary care paramedic: 21/33 (64%)
	Advanced care paramedic: 10/33 (30%)
Practice region	Lower Mainland: 13/33 (39%)
	Interior Health: 8/33 (24%)
	Island Health: 11/33 (33%)
	Northern Health: 1/33 (4%)

### The perceived roles and status of paramedic specialists and physicians in healthcare

The most prominent theme identified was that paramedics generally considered that physicians would theoretically be capable of providing a higher level of clinical advice than paramedic specialists. However, few were able to describe situations in which the paramedic specialists were unable to provide the required level of clinical advice or where the paramedic specialist escalated the consult to the available physician.

Every time I’ve talked to the physician I would have been just as happy to talk to a paramedic specialist (Participant 3A)

A number of paramedics stated they felt the title alone of physician was a gateway to additional services and pathways within the broader healthcare system which would not be afforded to paramedic specialists. Particularly, this was related to high-risk medicolegal situations as well as physician-level disagreements.

...there’s one thing unfortunately that is very hard to overcome is the power of a physician to physician conversation in our healthcare system...no matter how well you present yourself purely the title of having a doctor phone another doctor makes the wheels of the system work more effectively (Participant 2E)

Many paramedics suggested they would not consult a physician for many of the concerns they discussed with the paramedic specialists. Particularly, paramedics were more likely to call a paramedic specialist for considerations and general advice not mandatory within the clinical practice guidelines.

Paramedic specialists it’s a free for all, you can call them for anything. Any time you’re wondering anything, if you’re unsure, if you’re wondering if you should call for help we just call (Participant 3A)

Reasons for electing to consult a paramedic specialist when a physician would not be consulted included preparatory consultations prior to arriving on scene, support under stress, validation of clinical decision making, destination decision making and support on pediatric calls. Not only were the paramedic specialist able to provide remote support in these consultations, they were able to facilitate safer and more advanced out-of-hospital care.

When I was first coming out and dealing with pediatric populations, I liked to discuss dosing and routes of administration, just to kind of talk it out before I gave it on scene (Participant 4D)

### The influence of relationships and culture on clinical consultations

Given paramedic specialists are recruited from BCEHS and often have significant local experience, it is unsurprising paramedics made frequent mention of pre-existing relationships influencing clinical consultations. Each group made mention of how previous personal or professional relationships may contribute to their experience when accessing teleconsultations. This was particularly when compared against the lack of relationships with physician-based teleconsultations.

Having gotten to know a number of paramedic specialists in Vancouver, being able to phone and speak with someone who to me is a known quantity...and that actually really helps instead of just phoning “hello Dr Smith, who I’ve never met before”. And it shouldn’t matter, but it does. (Participant 3D)

### Practicalities of out of hospital care and the importance of lived experience

Although physicians employed in physician-delivered teleconsultations come from extensive backgrounds in emergency and critical care medicine, the extent to which they have experienced out-of-hospital is variable. Some will have practiced as paramedics, whilst others will only have theoretical knowledge. Although paramedics had described a theoretical possibility of physician providing higher level clinical advice, they discussed at length the importance of the paramedic specialists’ lived experience in promoting collaborative decision-making and providing effective advice.

I know they can imagine what’s actually happening when I describe it, because they’ve probably lived it and done it. So that’s really comforting. (Participant 5E)

…it’s a two-person crew doing CPR down like four flights of stairs. But they don’t understand that aspect of the job because they haven’t done it. (Participant 1C)

### Provision of appropriate clinical advice

The variety of paramedic license levels in BCEHS leads to variable clinical practice guidelines and mental models for the delivery of medical care. Paramedics identified that physician-based teleconsultations were less familiar with the clinical practice guidelines and scope of practice for each license level when compared against paramedic specialist colleagues. This on occasion was reported to delay patient care as they were required to explain their scope of practice prior to engaging in a clinical consultation. This was in contrast to the paramedic specialists, who were described to work from a shared mental model with an intimate knowledge of the various scopes of practice and who could tailor advice as such.

I’ve been on a forest road and had a physician ask me for troponin levels on a cardiac chest pain patient (Participant 2F)

When you speak with the paramedic specialists, I don’t have to explain as much. It’s perfectly okay for me to assume that when I say “hi, I’m X, I’m a primary care paramedic working in community Y” that they understand all of the things behind that (Participant 2C)

### Professional trust and respect

Professional trust and respect was identified to impact on the reception of teleconsultations and delivery of clinical advice. Paramedics described feeling they were required to convince physicians of their competence as a medical professional when requesting physician-delivered teleconsultations, before even commencing the clinical consultation. This was in comparison to experiences with paramedic-delivered teleconsultations, where paramedics reported they felt respected as competent professional colleagues.

If I’m talking to a physician, they will frequently ask questions that imply they’re second guessing my clinical judgement on scene...they will ask “are you sure they’re not breathing”, yes I’m sure. “But have you checked they’re not breathing?” (Participant 3D)

### Mentorship in out of hospital care

Paramedics identified that the paramedic specialists provided a degree of mentorship not experienced with physician-delivered teleconsultations. Primarily, this centred on the implementation of novel or unfamiliar clinical practice guidelines or skills. However, the paramedic specialists were also described to provide mentorship to paramedics dealing with morally or ethically challenging situations, which was not reported to be the case with physician-led teleconsultations. In addition, paramedics reported that the paramedic specialists would often take the time to call paramedics back following completion of the event to provide debriefing or real-time education.

This is a brutal job to start out in…having this paramedic specialist resource has made the learning curve a lot softer (Participant 3A)

When things bother me I do a debrief with them afterwards…it’s good. It didn’t go as well with physicians as it does certainly now with the paramedic specialists (Participant 5C)

### Clinical governance and education requirements

During deliberations about the future of paramedic- and physician-delivered teleconsultations paramedics discussed the need for clear role definitions, consistent education and clinical performance reporting as the role of paramedic-delivered teleconsultations was felt to be unclear during its initial deployment. Paramedics also described wishing to have access to various guidelines used by the paramedic specialists during consultations to better understand their role and decision-making framework.

There’s a real need to articulate a common training syllabus and educational pathway for online medical support and prehospital care...we’ve seen the development in nursing of the discipline of tele-nurse support...our approach in our training program is relatively new and still fairly ad hoc. (Participant 2E)

### The emergent theory

**Fig. 2:**
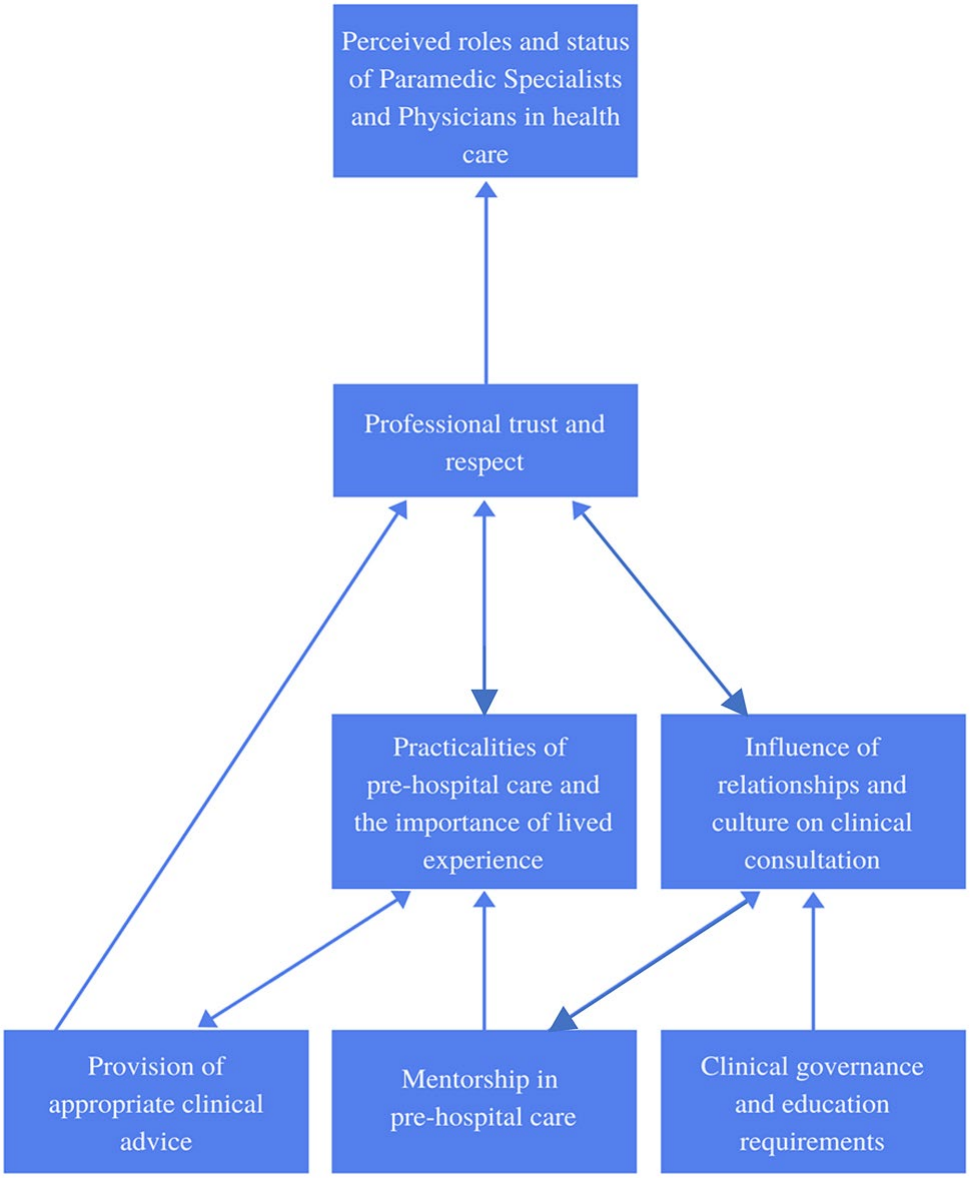
The emergent theory

Interpretive theories, more common in constructivist approaches to grounded theory, prioritize understanding rather than explanation and acknowledge that the theory rests on the theorist’s interpretation of the phenomenon [25]. When considering and interpreting the data from the focus groups, the theoretical model that emerged was *paramedics increasing ownership of their profession* (Fig. 2).

Since the introduction of CliniCall paramedics have seen a shift from heavy reliance on physicians for clinical support towards paramedic specialists predominating clinical consultations. Paramedics stated that paramedic specialists were consistently able to provide the level of clinical advice necessary without the need to escalate to physician teleconsultations. This shift in roles was underpinned by the professional trust and respect provided by the paramedic specialist in comparison with physicians, creating a supportive environment for consultations. Paramedics felt paramedic specialists were uniquely suited to provide teleconsultations in a way physicians were not based on their lived experience of out-of-hospital care and the associated complexities of logistical and clinical considerations. The peer-to-peer relationship established by the paramedic specialist group was facilitated by their pre-existing relationships, which in turn led to them functioning as mentors for paramedics around the province. This also appeared to establish an expectation of robust and transparent clinical governance and advanced education in paramedic leaders.

## Discussion

Paramedics clearly placed significant value on how the lived experience of the paramedic specialists allowed for them to more comprehensively understand the situation they were confronting and provide a mix of both logistical and clinical advice. In addition, paramedics compared their experiences with professional trust and respect during teleconsultations and recounted they frequently felt there was little trust or respect from physicians. The seemingly greater professional respect afforded by the paramedic specialists appeared to contribute to a supportive culture fostering consultation and shared-decision making, which in turn allowed the paramedic specialist group to facilitate safer and more advanced out-of-hospital care. Particularly, the perceived lack of professional respect appeared to translate into how respondents used their available teleconsultation services, with many paramedics suggesting they would not consult a physician for many of the concerns they discussed with the paramedic specialist group.

A large volume of paramedics in this research identified pre- and post-call discussions with paramedic specialists as a critical function of their role, as well as consultations on morally and ethically complex decisions such as those at the end of life or where there is ambiguity in the most appropriate course. Paramedics have previously identified the immediate aftermath of a traumatic patient encounter as a key period in how they process and make meaning from morally and ethically challenging situations [26], with rates of PTSD in paramedics amongst the highest of any emergency service [27]. Given these consultations were not commonly reported with physician-delivered teleconsultations, it is possible these provide a key safety net for a paramedic culture fraught with traumatic stressors, as well as complex moral and ethical decisions which must be made under significant time pressures.

### Limitations and recommendations

This research was conducted by a practicing advanced care paramedic with previous access to both physician and paramedic-delivered teleconsultations. Whilst strategies were used to improve academic rigor, it is possible residual bias was present. It is unlikely a sample of 33 paramedics in a single system represents a global perspective and so this research should be repeated in additional settings. Further investigations should be conducted into the how paramedic specialist consultation impacts patient outcomes, the optimal curriculum for paramedics delivering teleconsultations, as well as the perspectives of other key stakeholders.

## Conclusion

Paramedic practice is rapidly developing with a number of alternative roles for paramedics, including paramedic-delivered teleconsultations. This constructivist grounded theory investigation into paramedic perceptions of peer-to-peer teleconsultation produced the theory *paramedics increasing ownership of their profession*. Paramedics viewed paramedic-delivered teleconsultations positively and there was a strong desire for highly educated paramedics functioning in a system with sufficient clinical governance to provide the majority of teleconsultations. In light of the limitations of this study, Anglo-American paramedic system utilizing paramedic-delivered teleconsultations should consider additional investigations into this growing area of paramedic practice.

## Supplementary Information

Below is the link to the electronic supplementary material.

Supplementary file1 (DOCX 22 kb)

Supplementary file2 (DOCX 20 kb)
