# Supplementary material for: Paramedic-delivered teleconsultations: a grounded theory study
Source: CJEM. 2021 Dec 7;24(2):167–73. doi: 10.1007/s43678-021-00224-6 (PMC8904334; doi:10.1007/s43678-021-00224-6)
Supplement: Supplementary file 1 — Supplementary file1 (DOCX 22 kb) [file 43678_2021_224_MOESM1_ESM.docx]

**Appendix 1 – Focus Group Questions Guide**

***Introduction (10 minutes)***

Good morning/afternoon/evening and thank you for joining me for this session. My name is Richard Armour and I am both an Advanced Care Paramedic with the British Columbia Emergency Health Services (BCEHS), as well as a student at the University of Sheffield studying a Master’s of Science in Advanced Emergency Care. I am currently completing my dissertation as a component of my studies and have decided to investigate the novel role of paramedics in providing peer-to-peer online medical support.

This is an area which has seen a lot of international uptake and growth, with many ambulance services now utilizing paramedics in a variety of roles within the dispatch centre. In British Columbia, this program is more commonly referred to as the “CliniCall” service provided by Paramedic Specialists. Despite the widespread adoption of programs similar to CliniCall, there is scant research examining how paramedics feel about peer-to-peer support, particularly when compared to physician-led online medical support. This is what I am hoping to shed some light on with my research.

Before we begin I would like to acknowledge that involvement with CliniCall can often come under times of extreme stress and distress. If this discussion causes any emotional distress, please know this is a supportive and safe environment to raise this if you feel comfortable. Please know that you are, of course, able to stop your involvement with the discussion at any time. However, if this is the case I may pause the focus group briefly to help you ensure you are safe to travel before leaving the group. I have also left business cards for the Critical Incident Stress (CIS) team on the table, and encourage you to reach out to this service if required.

During this discussion there are no wrong answers and I fully expect a wide range of differing points of view. Please feel free to bring up your opinion even if it differs with the opinion of the group. I would like to encourage everyone to remain respectful in their discussions during times of disagreement, and that although I am interested in both positive and negative comments on the service there should be no discussion of specific Paramedic Specialists involved with the program.

As outlined in the confidentiality agreements, today’s session will be recorded by audio and video. The session is recorded as I am unable to write fast enough to record everything you will all say, and I have no interest in missing a single opinion you offer today. I will only use first names for identification and your data will be stored in accordance with Canadian federal regulations.

I think the easiest way to begin with some introductions, given you now know who I am! Please go around the group and let me know your first name, where you currently work, how long you have worked as a paramedic and what drove you to join the paramedic profession.

***Research Objective 1 (15 minutes)***

- Can you please elaborate on your organization’s criteria for engaging paramedic-led online medical support?
  - Are these suggestions, or mandatory consultations?
- Are you aware of the criteria Paramedic Specialists use to escalate discussions to physician-led online medical support?
  - If yes, please elaborate *(Prompt)*
  - If no, what particular clinical consultations do you believe should be managed in consultation with a physician rather than a Paramedic Specialist?
- Reflecting on your most recent engagements with paramedic-led online medical support, if the CliniCall service were unavailable to you would you have consulted physician-led online medical support?
  - Considering the consultations you have had with Paramedic Specialists, are there any times you have consulted with a Paramedic Specialist where you would not have considered a physician-led online medical support consultation?
  - What, if any, are the common characteristics of cases where you have consulted with a Paramedic Specialist where you may not have considered physician-led online medical support?
- If you were to work with a new paramedic in your service, how would you explain the difference between when you may call for Paramedic Specialist consultation compared with physician-led online medical support?
  - *Prompt – move beyond guidelines. Are there circumstances where consultation with either service happens outside the written guidelines?*

***Research Objective 2 & 4 (30 minutes)***

- Tell me about positive experiences you have had with paramedic-led online medical support
  - Take a minute to consider what particular factors made this a positive experience (feel free to write them down) and please share with the group
    - Prompt the group further with responses from other participants – examine whether these are common factors or uncommon anomalies
  - Is there any one particular factor which makes for a positive experience with paramedic-led online medical support?
- Tell me about disappointing experiences you have had with paramedic-led online medical support
  - Again, take a minute to consider what particular factors led to this being a disappointing experience and please share with the group
    - Prompt the group further with responses from other participants – examine whether these are common factors or uncommon anomalies
  - Is there any one particular factor which makes for a disappointing experience with paramedic-led online medical support?
- When taking into consideration all the consultations you have had with Paramedic Specialists, would you consider the experience to overall be positive or negative?
  - If you were able to change or improve one thing about paramedic-led online medical consultation, what would it be?

***Research Objective 3 (15 minutes)***

- Tell me about positive experiences you have had with physician-led online medical support
  - Take a minute to consider what particular factors made this a positive experience (feel free to write them down) and please share with the group
    - Prompt the group further with responses from other participants – examine whether these are common factors or uncommon anomalies
- Tell me about disappointing experiences you have had with physician-led online medical support
  - Again, take a minute to consider what particular factors led to this being a disappointing experience and please share with the group
    - Prompt the group further with responses from other participants – examine whether these are common factors or uncommon anomalies
- When taking into consideration all the consultations you have had with physician support, would you consider the experience to overall be positive or negative?
- When comparing your experience with Paramedic Specialists and physician-led online medical support, does one stand out as a more positive experience than the other?
  - What factors contribute to your opinion? *(Prompt)*
  - Are there certain elements each service is able to provide which the other is not?
  - In what ways would you like to see the relationship between paramedic-led and physician-led online medical support developed? Is there a role for expansion in either role?

***Conclusion (10 minutes)***

Thank you again for taking the time to participate in this research. This will conclude our time together. I would like to make sure you all have a copy of the participant information sheet, which includes my contact information if you need to contact me for any reason. I would also like to remind everyone again about the availability of CIS peers if any issue we have discussed today has caused any distress. Before we conclude, are there any questions for me?
