# Supplementary material for: Paramedic-delivered teleconsultations: a grounded theory study
Source: CJEM. 2021 Dec 7;24(2):167–73. doi: 10.1007/s43678-021-00224-6 (PMC8904334; doi:10.1007/s43678-021-00224-6)
Supplement: Supplementary file 2 — Supplementary file2 (DOCX 20 kb) [file 43678_2021_224_MOESM2_ESM.docx]

**Appendix 2 – Focussed Codes**

| Codes | References |
| --- | --- |
| Advanced care paramedics Preference for Greater Flexibility between paramedic specialist and the physician | 14 |
| Appropriate Guidance Requires Understanding of Both Clinical & Practical Considerations | 45 |
| Benefit to critical care paramedics paramedic specialist for Advanced care paramedics but not primary care paramedic | 10 |
| Broad Criteria to Engage paramedic specialist | 11 |
| Broader Community Culture Influences Perceptions of paramedic specialist | 10 |
| Changing Culture from Decision-Making in Isolation to Shared Decision Making | 8 |
| Community Encouragement Increases Engagement and Positive Experiences | 2 |
| Consulting a Peer (paramedic specialist) is of Benefit | 29 |
| Consulting paramedic specialist Outside of Mandatory Indications | 7 |
| Decision About paramedic specialist v the physician Not the Decision of Paramedic | 5 |
| Desire for the physician to Maintain more Involvement with Pre-Hospital Care | 10 |
| Desire for paramedic specialist to Take on Larger Role & Responsibilities | 33 |
| Desire to See Establishment of Clear Subspecialty Program | 15 |
| Difficult to compare paramedic specialist & the physician because of differences in services provided | 5 |
| Difficulties Obtaining Critical Advice Leading to Moral Injury | 16 |
| Effective the physician Physicians Have Professional Respect for Paramedics | 2 |
| the physician Functions Well when Communication Well Structured | 6 |
| the physician is Perceived as an Authority Figure | 14 |
| the physician Less Familiar with Paramedic Scopes of Practice & Guidelines | 36 |
| the physician May Defer Indicated Treatments if Close to Hospital | 10 |
| the physician On-Call Model Divides Attention | 23 |
| the physician Providing Outdated Guidance of Guidance Contrary to Organizational Policy | 2 |
| Familiarity with Advanced care paramedics & critical care paramedics Influences Willingness to Consult | 3 |
| Initial Perception that paramedic specialist Was to Check Up on Paramedics Rather than Support Clinical Decision Making | 7 |
| Integration of virtual health into program seen as area for development | 4 |
| Lack of Clarity of Role & Consistent Guidelines | 28 |
| Lack of Relationship May Contribute to Poor Perception of paramedic specialist | 4 |
| Lived Experiences of Pre-Hospital Care Informing Positive Experiences | 44 |
| Mentoring Solo, Rural & Remote Practitioners | 16 |
| Narrow Indications for the physician Compared with paramedic specialist | 33 |
| Negative Experiences of paramedic specialist When Not Given Professional Respect | 7 |
| primary care paramedic Less Concerned with Differentiating Between the physician & paramedic specialist | 3 |
| Perception is Paramedics must Convey Competence to the physician in Addition to Clinical Discussion | 43 |
| Perception there is No Need for Relationships | 8 |
| Physician Considered Highest Level of Clinical Support | 45 |
| Positive Experience with paramedic specialist Influences Likelihood of Calling Again | 3 |
| Previous Negative Relationships May Influence Consultations | 4 |
| Previous Positive Relationship with paramedic specialist Considered Beneficial | 42 |
| Professional Relationship Superceding Personal Relationships | 3 |
| Provision of Support for Novel Clinical Procedures & Guidelines | 6 |
| paramedic specialist & the physician Unwilling to Pre-Approve Interventions to Minimize Time Away from Patient | 6 |
| paramedic specialist Able to Provide Scope-Appropriate Advice Better than the physician | 24 |
| paramedic specialist Facilitating Advanced Pre-Hopsital Care & Patient Safety | 10 |
| paramedic specialist Fostering Culture of Team-Based Care | 17 |
| paramedic specialist Fostering Shared Decision Making with all Clinical Levels of Staff | 26 |
| paramedic specialist Have a Shared Mental Model with Paramedics | 11 |
| paramedic specialist Have Greater Professional Respect for Paramedics | 12 |
| paramedic specialist Maintain Situational Awareness and Provide Advice in Context | 4 |
| paramedic specialist Maintaining Involvement & Providing Real-Time Education | 21 |
| paramedic specialist May Disagree with Organizational Direction | 3 |
| paramedic specialist May Have Limited Knowledge of Provincial Health Authority Guidelines | 13 |
| paramedic specialist Mediating Disagreements between Paramedics and Other Health Practitioners | 2 |
| paramedic specialist Past Experiences Influence Consultations | 1 |
| paramedic specialist program improved by moving into other centres | 1 |
| paramedic specialist Provide Advice which Paramedics will have to Explain to Other Clinical Staff | 4 |
| paramedic specialist Share Responsibility | 6 |
| paramedic specialist Support on Pediatric Calls | 8 |
| paramedic specialist Supporting Morally & Ethically Challenging Decisions | 12 |
| paramedic specialist Supporting Transfer & Destination Decision Making | 14 |
| paramedic specialist Used as Support Under Stress | 15 |
| paramedic specialist Used to Prepare for Arrival to Scene | 16 |
| paramedic specialist Validate Clinical Decision Making | 32 |
| paramedic specialist with Expertise in High Risk Hazards Advice | 2 |
| Recognition there is a Time & Place for Higher Clinical Advice | 4 |
| Supporting New Practitioner Transition to Practice | 15 |
| The Need for Independent Oversight | 12 |
| Transparency Considered Critical to Success of Program | 14 |
| Using paramedic specialist Program as Clinical Governance | 6 |
| Using paramedic specialist to support less frequently used guidelines | 2 |
| Would have Called the physician even if CliniCall wasn't available | 5 |
| Would not Call the physician for Same Reasons as paramedic specialist | 30 |
